# Green credit and market expansion strategy of high pollution enterprises—Evidence from China

**DOI:** 10.1371/journal.pone.0279421

**Published:** 2022-12-30

**Authors:** Qian Zhong, Xuemeng Ding, Xiaoke Sun, Hairui Zhao

**Affiliations:** 1 Department of Financial Engineering, School of Finance, Guangdong University of Foreign Studies, Guangzhou, Guangdong, China; 2 Department of Public Administration, School of Humanities and Laws, Hebei University of Technology, Tianjin, Hebei, China; 3 Department of Insurance, School of Finance, Guangdong University of Foreign Studies, Guangzhou, Guangdong, China; 4 Department of Finance, School of Economics and Law, University of Science and Technology Liaoning, Anshan, Liaoning, China; DePaul University, UNITED STATES

## Abstract

This paper uses the Difference-in-Differences method to test the impact of the promulgation of Green Credit Guidelines, a market-oriented environmental regulation, on the enterprise market expansion strategy, based on the panel data of Chinese A-share listed companies from 2008 to 2015. We find that the promulgation of Green Credit Guidelines significantly inhibited the market expansion strategy of high pollution enterprises. Two channels through which the Green Credit Guidelines affect the market expansion strategies of high polluters are increasing the cost of financing and promoting green R&D. Heterogeneity analysis finds that the impact of Green Credit Guidelines on the market expansion of highly polluting enterprises is more significant in non-state-owned enterprises and enterprises without equity incentive. Further analysis shows that the promulgation of Green Credit Guidelines damages the corporate image and profitability of high polluting enterprises, but it doesn’t increase the risk of high polluting enterprises. The results of this research could help relevant government departments to formulate practical environmental regulations and promote sustainable economic development.

## 1. Introduction

Over the past decade, China’s contribution to world economic growth has exceeded 30%, ranking first in the world. However, the long-term industrial development model of high investment, high consumption and high pollution has led to a series of problems such as excessive consumption of resources and environmental degradation, which has seriously affected the sustainable and healthy development of economy and social harmony [1]. As the largest carbon emitter, China’s electricity consumption is still dominated by traditional energy generation, with thermal power generation contributing more than 70%. Chinese government has actively explored an effective model for green economic development but has not fundamentally reversed China’s development model of high energy consumption and high emission [2]. The key question is whether China can develop appropriate environmental regulations to align firms’ profit maximization strategy choices with the country’s environmental protection strategy choices. In other words, whether there is an appropriate environmental regulation that makes Porter’s hypothesis valid.

In essence, green finance is a way to guide funds from the pollution field to the green field with the help of the effective allocation of financial resources, so as to realize the sustainable development of ecological environment and economy. Green credit is the main form of green finance in China, which is a bank dominated economy. Since 2007, the Chinese government has been building a green credit policy system and started small-scale pilot projects. In 2012, the China Banking Regulatory Commission issued Green Credit Guidelines, marking the full implementation of green credit policies in China. In recent years, China’s major banking institutions have gradually intensified their implementation of green credit. By the end of the first quarter of 2021, the green balance of 21 major Chinese banks reached 12.5 trillion yuan, ranking first in the world. The non-performing loan ratio of green credit remaining below 0.7% in the past five years, much lower than the overall non-performing level of various loans in the same period. China’s green credit policies are already bearing fruit, and commercial banks are supporting the upgrading of industrial structure and optimizing the allocation of resources through funds. The green credit policies strongly promote the transformation and development of China’s economy.

The enterprises, as for-profit organizations, aim to maximize profits. Green credit, a market-based environmental regulation, can only guide high-polluting enterprises to make a green transition by adjusting the amount of funds allocated among different enterprises [3]. However, Chinese high polluters are accustomed to relying on rent-seeking to obtain monopolistic positions in their industries [4, 5]. The differential allocation policy of green credit may intensify the rent-seeking behavior of high-polluting enterprises and make the market strategy choice of high-polluting enterprises contradict with the government’s sustainable development strategy choice.

That such a scenario is logically possible is by now beyond doubt. That such a mechanism is of quantitative importance, however, remains an empirical question. Unfortunately, the ‘micro’ observation that green credit reduces bank lending to high-polluting companies [3] and promotes innovation in high-polluting companies [5] do not constitute sufficient evidence of this mechanism.

Instead, we examine this mechanism at a relatively macro level by examining the behavior of enterprise market expansion strategy selection. Specifically, this paper takes the promulgation of Green Credit Guidelines in 2012 as a quasi-natural experiment, we assemble a firm-level dataset including 7272 samples from 2008 to 2015 and employ the difference-in-differences (DID) approach to identify causality.

We find that high polluting firms reduce the rate of market expansion after green credit guidelines are issued. This implies that even if the government adopts non-coercive environmental regulations, high polluters still adjust their corporate strategies to match the national strategic needs. This paper also provides empirical evidence from China for the validity of Porter’s hypothesis. We are interested in the channels through which green credit guidelines influence the strategic choices of high-polluting firms. We find that the Green Credit Guidelines increase the financing constraints and promote green R&D of high-polluting firms, all of which may inhibit high-polluting firms from adopting market-expanding strategies. We find that the Guidelines would be more influential if the firm is non-SOE or does not have equity incentives.

At the same time, we also found that although the green credit guidelines reduced the profitability of highly polluting enterprises, they did not increase the risk of highly polluting enterprises. This shows that a well-designed policy, such as the Green Credit Guidelines, can realize the green transformation of the economy on the premise of stabilizing market risk.

This paper contributes to our understanding of the role of the Green Credit Guidelines. We study the impact of green credit policy on the company from the perspective of enterprise strategic deployment. The existing literature mainly investigates the impact of green credit policy on corporate behavior at specific levels [6, 7]. This paper focuses on the choice of corporate strategy in the strategic management theory. We investigate the impact of market-oriented environmental regulation on the company’s strategic choice, reveal the internal relationship between environmental protection policy and company behavior from a deeper level, and enrich the research in the field of economic consequences of environmental protection policy. At the same time, this paper is also a beneficial attempt to the interdisciplinary research of finance, management and environmental protection.

This paper links the research on the influencing factors of the company’s strategic choice with the national strategy of environmental protection. The external environment and the company’s strategic choice have been discussed for a long time. Existing literature has focused on the impact of political, economic, institutional and other macro-environments as well as the industrial environment on the company’s strategic choices. This article takes the promulgation of China’s Green Credit Guidelines as the starting point, and extends the research on the factors affecting the company’s strategic choices to the fields of environmental protection and world sustainable development.

## 2. Institutional background

As early as the beginning of reform and opening up, the Chinese government has paid attention to environmental protection issues. In 1995, the People’s Bank of China issued the "Notice on Issues Related to Implementing Credit Policies and Strengthening Environmental Protection Work". The Notice requires financial institutions to include the environmental friendliness of enterprises in credit review conditions. This is China’s first document on green credit policies. However, the primary development goal at that time was economic growth, the contradiction between economic growth and environmental protection was not yet prominent. The green credit was not given enough attention. In 2007, the People’s Bank of China and the environmental protection department jointly issued the "Opinions on Implementing Environmental Protection Policies and Regulations to Prevent Credit Risks". The Opinions clearly stated that commercial banks should support environmental protection and control credit to polluting enterprises as an important part of fulfilling their social responsibilities. However, the green credit policy has not been effectively implemented, due to the lack of corresponding supporting measures and supervision mechanisms.

On February 24, 2012, the China Banking Regulatory Commission issued the "Green Credit Guidelines". The Guidelines make specific arrangements for the green credit business of banking financial institutions and the supervision of regulators from six aspects: organization management, policy and system construction, process management, internal control management and information disclosure, supervision and inspection. The Green Credit Guidelines are applicable to policy banks, commercial banks, rural cooperative banks, and rural credit cooperatives legally established in China. The Guidelines require banking financial institutions to formulate their own green credit policies in order to strengthen the management of enterprises’ environmental and social responsibility. The Guidelines also ask banking financial institutions to invest more in key areas such as green economy, low-carbon economy and circular economy. The Guidelines clearly states that the regulatory authorities must comprehensively evaluate the green credit performance of banking financial institutions, and take the evaluation results as an important basis for the regulatory rating, institutional access, business access and senior executives’ performance evaluation of banking financial institutions in accordance with relevant laws and regulations. The "Green Credit Guidelines" is China’s first normative document specifically aimed at green credit policies. It defines the framework of China’s green credit policy system and is of great significance to the development of green credit and green finance.

## 3. Literature review and theoretical analysis

### 3.1 Literature review

More and more studies have found that environmental pollution will affect climate change and human health [8–11], so countries have promulgated policies to promote sustainable development. The promulgation of the Green Credit Guidelines in 2012 provided practical guidance for Chinese financial institutions on how to effectively carry out green credit and support the establishment of a low-carbon circular development industrial system. So far, a large number of literatures have carried out research on the effect of green credit. The existing literature mainly focuses on the following two aspects: One is to analyze the risks and uncertainties of green credit from the macro level. For example, Shen and Ma [12] believe that the promotion of green credit must solve the contradiction between environmental protection and the maximization of GDP performance. Zhao et al. [13] find that the relationship between environmental regulation and green total factor productivity is complex, which is reflected in the differences and nonlinearity between cities with different monitoring levels and different economic development levels. Liu et al. [14] calculate the total factor productivity of the green economy and find that green finance promotes high-quality economic development through technological innovation and industrial structure upgrading. The other is to analyze the effect of green credit policy implementation from a micro level. For example, Liu et al. [15] finds that green credit policy can help reduce the output of papermaking and chemical enterprises in the short to medium term by using CGE model. Wang et al. [7] use the DID model to find that green credit policies inhibit the financing of high-polluting companies. Much of the existing literature focuses on the impact of Green Credit Guidelines on corporate financing behavior, investment behavior, and R&D behavior at the micro level. But all these behaviors are only a slice of the corporate strategy. The existing literature does not sufficiently explain what impact the Guidelines will have on the strategic choices of high polluting firms.

In management, there are many ways to divide corporate strategy. Among them, the classification method of Miles and Snow [16] can cover other mainstream strategic classifications and is easy to measure. The classification method of Miles and Snow [16] is widely accepted by scholars as a result. According to the definition of Miles and Snow [16], corporate strategy is divided into offensive, analytical and defensive. Companies implementing different strategies have significant differences in business models and organizational structures. Offensive companies are keen on product creation, technology research, and market development. The offensive company’s management team changes frequently and the employee turnover rate is relatively high. Unlike offensive companies, defensive companies usually focus on several existing products or services, and pay more attention to how to reduce product costs and improve production efficiency, lack of enthusiasm for new business or new market expansion. The management team in defensive companies is more stable, and the average tenure of employees is relatively long. The characteristics of analytical companies are somewhere in between.

In fact, the company’s strategy is neither good nor bad, only suitable or not [16]. If the company wants to achieve sustainable development, it needs to make its own strategic choices. The external environment is an important factor affecting the company’s strategic choice. Chandler [17] first proposes that the company cannot change the external environment, and its strategy must adapt to the current situation and change trend of the external environment. Learned et al. [18] further points out on the basis of Chandler [16] that the process of strategic selection is essentially a process of matching the company’s internal characteristics with the external environment, and proposes the SWOT model. Porter [19] put forward the theory of competitive strategy, holding that the industrial structure is the key factor determining the company’s profitability. Institutional theory (Meyer and Rowan [20]) also believes that organizational behavior actively adapts to the external institutional environment, so the company’s strategic choice will also be affected by the institutional environment.

China’s 14th Five-Year Plan puts forward the long-term goal of basically realizing socialist modernization by 2035: widely forming a green production and lifestyle, steadily decreasing carbon emissions after reaching the peak, fundamentally improving the ecological environment, and basically realizing the goal of building a beautiful China. It shows that environmental protection and sustainable development have been highly valued by the Chinese government. Therefore, environmental protection is also an external environmental factor that cannot be ignored when the management chooses the company’s strategy in recent years.

### 3.2 Theoretical analysis

Rational enterprises will make discretionary choices according to their own conditions, facing the constraints brought by market-oriented environmental regulation. The self-selection behavior of enterprises is heterogeneous: reduce production scale or even withdraw from the market, upgrade products to reduce pollution or transform [21]. Due to financial and technical constraints, small enterprises cannot internalize the costs caused by environmental regulation. Shutdown has become a common means to deal with environmental regulation for small enterprises. The enterprises, with a certain capital and technological foundation, may adjust their production lines and actively innovate technology to achieve energy conservation, emission reduction and industrial upgrading. The promulgation of green credit guidelines shows the Chinese government’s determination to develop green finance and govern the environment. The Guidelines strengthens the bank’s supervision of highly polluting enterprises, increases the probability of negative information from high-polluting companies being exposed, and restrains the occurrence of opportunism [22]. All in all, the promulgation of the Green Credit Guidelines, as a market-oriented environmental regulation, will change the survival mode of high polluting enterprises—making profits by polluting the environment. At this time, the company’s management needs to review the situation and make adjustments to its strategic choices to fit the current China’s national strategy of achieving a beautiful China. Regardless of whether companies choose to shut down high-polluting industries or carry out green upgrades to the industrial chain, they will not adopt a radical market expansion strategy in the short term. Therefore, we propose the following hypothesis:

**Hypothesis 1 (H1):** *The promulgation of the Green Credit Guidelines inhibits the market expansion strategy of high-polluting companies*.

According to the green credit guidelines, commercial banks strictly control the credit threshold and take the environmental compliance of enterprises as an important condition for granting loans. The supply of credit to highly polluting enterprises decreased. The financing cost of highly polluting enterprises increased, and bank loans, especially long-term loans, decreased significantly [23, 24]. Modigliani Miller theory [25, 26] points out that the company value depends on the company’s investment decision, and has nothing to do with the financing decision and capital structure. However, in actual business activities, enterprises often have over investment and under investment [27]. The existing literature find that enterprises can supplement free cash flow through debt financing [28], overinvest with free cash flow [29, 30]. Environmental protection regulation reduces the over investment of high pollution enterprises by limiting the financing capacity [31]. In other words, after the promulgation of the green credit guidelines, the financing costs of high polluting enterprises have increased, over investment has decreased, and the market expansion strategy has become defensive. Therefore, we propose the following hypothesis:

**Hypothesis 2 (H2):** *The promulgation of Green Credit Guidelines inhibits the market expansion of high polluting enterprises by increasing the financing cost*.

As a market-based environmental regulation, the Green Credit Guidelines can provide more flexible and effective innovation incentives [32, 33]. The Green Credit Guidelines require banks to regularly conduct internal control inspections and evaluations of green concepts such as environmental protection, conservation, and sustainable development on loans. The Guidelines also require banks to provide preferential lending policies to companies that meet the conditions for environmental protection and sustainable development. In order to obtain the economic benefits of preferential loans, companies seeking to maximize profits will increase the R & D of green innovative technologies. Green innovation can bring new competitive advantages to enterprises, because green environmental protection and sustainable development are advocated all over the world [34, 35]. For companies with strong innovation capabilities, their innovation activities often have the characteristics of sustainability. The innovation activities of enterprises with strong innovation ability are often sustainable. Enterprises with relatively weak innovation ability are more vulnerable to external factors such as subsidies and rewards due to limited relevant innovation resources [36]. All in all, the promulgation of the Green Credit Guidelines will guide companies to increase green innovation R & D. Technology research and development business enhance the company’s growth, but also increase the company’s performance risk [37, 38]. To reduce the uncertainty of the company in the future, companies that increase R & D may reduce the scale of market expansion in the short term, that is, the R & D tendency will crowd out the company’s market expansion tendency. Based on this, we propose following hypothesis:

**Hypothesis 3 (H3):** *The promulgation of Green Credit Guidelines inhibits enterprise market expansion by encouraging green R & D of highly polluting enterprises*.

## 4. Methods

### 4.1. Data source

This paper takes Chinese A-share listed companies from 2008 to 2015 as research samples, and the data required are from the CSMAR database. In this paper, the samples are processed as follows: 1. Eliminate the sample of financial listed companies; 2. Eliminate the sample of companies that have been listed for less than five years, because we need five years of financial data to calculate the company’s market expansion tendency. 3. Eliminate the samples with missing data in variables. 4. Eliminate companies with a leverage ratio of more than 1. 5. Winsorize the continuous variables at the 1% level.

The promulgation of the Green Credit Guidelines is a completely exogenous event for enterprises. According to the specific content of the Green Credit Guidelines, the Guidelines only affect the credit policies and corporate social responsibilities of high-polluting companies. Therefore, we take high-polluting companies as the treated group and other companies as the control group, use the DID (Difference-in-Difference) model to analyze the impact of green credit policies on corporate expansion strategies. We consider these companies as high-polluting companies: companies in the 14 high-polluting industries disclosed in the "List of Listed Companies Environmental Inspection Industry Classification Management Directory" issued by the Ministry of Environmental Protection in June 2008. The 14 heavily polluting industries are: thermal power, steel, cement, electrolytic aluminum, coal, metallurgy, building materials, mining, chemicals, petrochemicals, pharmaceuticals, light industry, textiles and tanning. We use the "List of Listed Companies Environmental Inspection Industry Classification Management Directory" promulgated in June 2008 for the following reasons: First, this is the classification issued by the Ministry of Environmental Protection of China, which is authoritative. Second, this is a classification issued before the Green Credit Guidelines and before the sample research time of this article. Using this classification can effectively solve the problem of sample selection.

We finally identified 7272 enterprise-annual samples, including 1032 high-polluting enterprise samples and 6,240 control group samples.

### 4.2. Model setting and definition of variables

We use a Difference-in-Differences model to test the influence of Green Credit Guidelines on enterprises’ market expansion strategy, following Liu et al. (2019). A standard Difference-in-Differences (DID) model is as follows: This section may be divided by subheadings. It should provide a concise and precise description of the experimental results, their interpretation, as well as the experimental conclusions that can be drawn.

$$y_{i,t}=\alpha_{0}+\alpha_{1}du+\alpha_{2}dt+\alpha_{3}(du\times dt)+\alpha_{4}control+\varepsilon_{i,t}$$ (1)

*du* is a dummy variable to measure whether enterprises are affected by policies. If firm i is affected by the policy *du* = 1, otherwise *du* = 0. *dt* is a dummy variable representing whether the policy is implemented. *dt* is 0 before policy is implemented and 1 after policy is implemented. *ε*_*i*,*t*_ is the error term, which is assumed to be normally distributed with zero mean value and constant variance according to the existing literature [39–41].

When we use the bidirectional fixed effects DID model, *du* will be absorbed by individual fixed effect, *dt* will be absorbed by the time fixed effect. Therefore, the DID model is set as follows:

$$Strategy_{i,t}=\beta_{0}+\beta_{1}(Pollute_{i}\times Policy_{t})+\beta_{2}control+\lambda_{i}+\upsilon_{t}+\varepsilon_{i,t}$$ (2)

*Strategy* is the variable we use to measure the company’s market expansion strategy. Referring to the existing literature [42, 43], we use this method to measure the company’s market expansion strategy. In the first step, we calculate the ratio of the sum of the company’s sales expenses and management expenses to the operating revenue as the proxy index of the company’s market expansion tendency. In the second step, we calculate the average value of the company’s market expansion tendency in the past five years and sort it into six groups from small to large by industry-year. Assign 1 to the minimum value group, 2 to the secondary small value group…… and 6 to the maximum value group. The higher the score, the more offensive the company’s expansion strategy.

Compared with the method of directly using financial indicators, this method has the following advantages: 1. This indicator covers the company’s resource allocation and operating results in the past five years, and reflects the company’s long-term action plan and implemented strategy. 2. Using this indicator can avoid the possible endogenous problems caused by the direct use of financial indicators. 3. We can calculate the corporate strategy of each company, which is conducive to large sample empirical research. In the robustness test part, we also use the market position in the next period and the growth rate of sales scale in the next period to measure the enterprise’s market expansion strategy.

*Pollute* × *Policy* is the main explanatory variable in the Difference-in-Difference model. *Pollute* is a virtual variable that indicates whether the company is high-polluting. *Pollute* = 1 when a company is high-polluting, otherwise *Pollute* = 0. *Policy* is a virtual variable that measures the promulgation of Green Credit Guidelines. *Policy* = 0 if samples are in 2008 to 2011, *Policy* = 1 if samples are in 2012 to 2015. The coefficient of *Pollute* × *Policy* measures the net impact of Green Credit Guidelines on the market expansion strategy choice of high-pollution enterprises.

*control* is the control variable. We control the following main characteristics that may affect corporate strategy in Formula 2: company size (*size*), leverage (*leverage*), return on equity (*ROE*), listing years (*age*), state owned enterprise (*SOE*), ownership concentration (*Top5Hold*) and industry position (*Indpos*). In addition, corporate strategic decisions are mainly decided by the board of directors and the management. Therefore, we control the variables related to the characteristics of the board of directors and management: board size (*Board*), proportion of independent directors (*Indep*) and mean age of the management (*MgAge*).

See Table 1 for a description of the variables.

**Table 1:** Descriptions of variables.

Variable	Description
*Pollute*	1 for highly polluting enterprises, 0 for non-highly polluting enterprises
*Policy*	0 for policies issued during 2008–2011, 1 for policies issued during 2012–2015
*Strategy*	Score on the company’s market expansion tendency. Calculate the ratio of the sum of the company’s sales expenses and administrative expenses to the company’s operating revenue as the company’s market expansion tendency. Calculate the average value of the company’s market expansion tendency in the past five years, sort the values from small to large and divide them into 6 groups by industry-year. Assign 1 to the minimum value group, 2 to the secondary small value group, and so on, and 6 to the maximum group.
*size*	The natural logarithm of the enterprise’s assets at the end of the year
*age*	The year of the current year minus the year of listing.
*leverage*	Total liabilities/total assets.
*ROE*	Net income/equity.
*SOE*	1 for a state-owned enterprise, 0 for others.
*Top5Hold*	Shareholding ratio of the top five shareholders.
*board*	Number of directors
*Indep*	Number of independent directors / number of board members
*MgAge*	Average age of non-independent directors and executives
*Indpos*	Operating income of the company / operating income of the whole industry

## 5. Results and analysis

### 5.1 Descriptive statistics

Table 2 reports the descriptive statistical results of the main variables. The average value of strategy is slightly larger than the median, indicating that there are certain companies with a strong tendency to expand. The standard deviation of strategy is 1.347, indicating that different companies have different market expansion propensities. The standard deviation of size is 1.226, indicating that the size of the assets of each enterprise is quite different. The mean of age is 12.17, indicating that most listed companies have been established for more than 10 years. The mean of lev is 0.504, indicating that most companies have low debt ratios. The average soe value is 0.595, indicating that China’s state-owned enterprises are slightly more than non-state-owned enterprises. The median of Top5hold is 49.79, indicating that nearly half of the companies’ top five shareholders hold more than 50% of the shares. The mean of board is 9.068, indicating that the average number of shareholders of listed companies is 9. The mean of MgAge is 47.8, indicating that most of the directors and executives of listed companies are middle-aged. The median of Indpos is 0.013 and the average is 0.079, indicating that most industries in China are close to a perfectly competitive market.

**Table 2:** Descriptive statistics.

variable	N	mean	p50	sd	min	max
*Strategy*	7272	3.043	3.000	1.344	1.000	6.000
*size*	7272	22.288	22.150	1.226	18.831	25.827
*age*	7272	12.171	12.000	4.594	5.000	23.000
*leverage*	7272	0.504	0.512	0.195	0.073	0.993
*ROE*	7272	0.077	0.076	0.138	-1.033	0.570
*SOE*	7272	0.595	1.000	0.491	0.000	1.000
*Top5Hold*	7272	49.832	49.790	15.271	16.920	86.320
*board*	7272	9.068	9.000	1.815	5.000	15.000
*Indep*	7272	0.368	0.333	0.053	0.250	0.571
*MgAge*	7272	47.848	47.944	3.075	39.273	55.133
*Indpos*	7272	0.079	0.013	0.221	0.000	1.529

Note: All monetary terms are denominated in Chinese Yuan (CNY). p50 refers to the 50% quantile, sd represents the standard deviation, max and min represent the maximum and minimum values respectively.

Table 3 shows the differences between the treated group and the control group before and after the policy is promulgated. We can see from the table that the mean value of *Strategy* in high polluting enterprises after the promulgation is 2.769, which is significantly lower than 3.126, the mean value before the promulgation. This shows that high polluting enterprises have significantly reduced the market expansion tendency after the promulgation of Green Credit Guidelines. However, there is no significant difference in the *Strategy* of non-highly polluting companies before and after the promulgation of the policy, only from 3.037 to 3.085. So non-highly polluting enterprises have a slight tendency of market expansion, but the change is not significant. The above information shows that the promulgation of the Green Credit Policy does have a negative impact on the market expansion strategy of the experimental group companies. This impact needs to be further studied using the DID model.

**Table 3:** Two-group testing of the difference of the means.

	Treated			Control		
	Before	After	MeanDiff	Before	After	MeanDiff
*Strategy*	3.126	2.769	0.357***	3.037	3.085	-0.048
*size*	22.290	22.675	-0.385***	22.166	22.429	-0.263***
*age*	11.311	13.055	-1.744***	12.147	12.645	-0.498***
*leverage*	0.519	0.478	0.041***	0.519	0.467	0.052***
*ROE*	0.075	0.028	0.047***	0.088	0.071	0.016***
*SOE*	0.680	0.588	0.092***	0.631	0.444	0.187***
*Top5Hold*	49.761	52.346	-2.585***	49.409	49.974	-0.565
*board*	9.324	8.968	0.356***	9.134	8.737	0.397***
*Indep*	0.363	0.372	-0.010***	0.367	0.372	-0.005***
*MgAge*	47.731	49.060	-1.329***	47.557	48.188	-0.632***
*Indpos*	0.038	0.054	-0.016*	0.083	0.116	-0.033***

Note:

***, ** and * represent significance levels of 1%, 5% and 10%, respectively. MeanDiff represents the difference of means.

### 5.2. Analysis of regression results

To verify Hypothesis 1: Green Credit Guidelines will inhibit the market expansion strategy of high-polluting companies, we conduct an empirical study according to Formula 2. The regression results are shown in Table 4. The first column is the two-way fixed effect model with only *Pollute* × *Policy*. The coefficient of *Pollute* × *Policy* is -0.2453, statistically significant at the 1% level. This shows that the promulgation of the Green Credit Guidelines has significantly inhibited the market expansion strategy of high-polluting companies. Hypothesis 1 holds. The green credit policy is an effective environmental protection policy. The second to fourth columns are the regression results after adding other control variables, the coefficient of *Pollute* × *Policy* is still significantly negative.

**Table 4:** Green Credit Guide and enterprise market expansion strategy.

	(1)	(2)	(3)	(4)
*Pollute*×*Policy*	-0.2453***	-0.1229**	-0.1630***	-0.1638***
	(0.064)	(0.062)	(0.062)	(0.062)
*size*		0.5176***	0.4470***	0.4509***
		(0.042)	(0.045)	(0.045)
*age*		-1.1952***	-1.0016***	-0.9577***
		(0.267)	(0.268)	(0.268)
*leverage*		0.8297***	0.9685***	0.9530***
		(0.157)	(0.157)	(0.157)
*ROE*		1.4472***	1.3990***	1.3905***
		(0.117)	(0.117)	(0.117)
*SOE*			-0.0767	-0.0600
			(0.117)	(0.117)
*Top5Hold*			0.0162***	0.0159***
			(0.002)	(0.002)
*board*			0.0090	0.0079
			(0.015)	(0.016)
*Indpos*			-0.5110**	-0.4912**
			(0.206)	(0.206)
*Indep*				-0.2077
				(0.400)
*Mgage*				-0.0360***
				(0.011)
*Constant*	3.3308***	1.4134	0.4498	1.7656
	(0.042)	(2.379)	(2.383)	(2.417)
*Observations*	7,272	7,272	7,272	7,272
*R-squared*	0.037	0.108	0.117	0.119
*Number of stkcd*	1,727	1,727	1,727	1,727
*Firm*&*Year*	YES	YES	YES	YES

Note:

***, ** and * represent significance levels of 1%, 5% and 10%, respectively.

We can also find from the regression results that the coefficient of *size* is significantly positive, indicating that the larger the scale of the company, the more motivated it is to adopt a radical market expansion strategy. The coefficient of *age* is significantly negative, which means that the younger the company is, the more likely it is to adopt aggressive expansion strategies. There is a significant positive correlation between *leverage* and *Strategy*, which means that external financing is an important source of funds for the market expansion of the Chinese enterprise. The more profitable companies are, the more motivated they are to adopt aggressive strategies, because *ROE* and *Strategy* are significantly positively correlated. The *Top5hold*’s coefficient is significantly positive, that is, a company with a higher degree of equity concentration is more likely to aggressively expand the market. Interestingly, the negative coefficient of *MgAge* tells us that the older the managements, the more defensive a company’s business strategy, which may be related to the calm and moderate personality of the Chinese elders. *Indpos* and *Strategy* are significantly negatively correlated, indicating that companies with a higher market share tend to be more prudent in operation, while companies with a lower market share have a strong tendency to market expansion. The regression results in Table 4 are consistent with the actual situation.

### 5.3 Parallel trend test and placebo test

**Fig 1:**
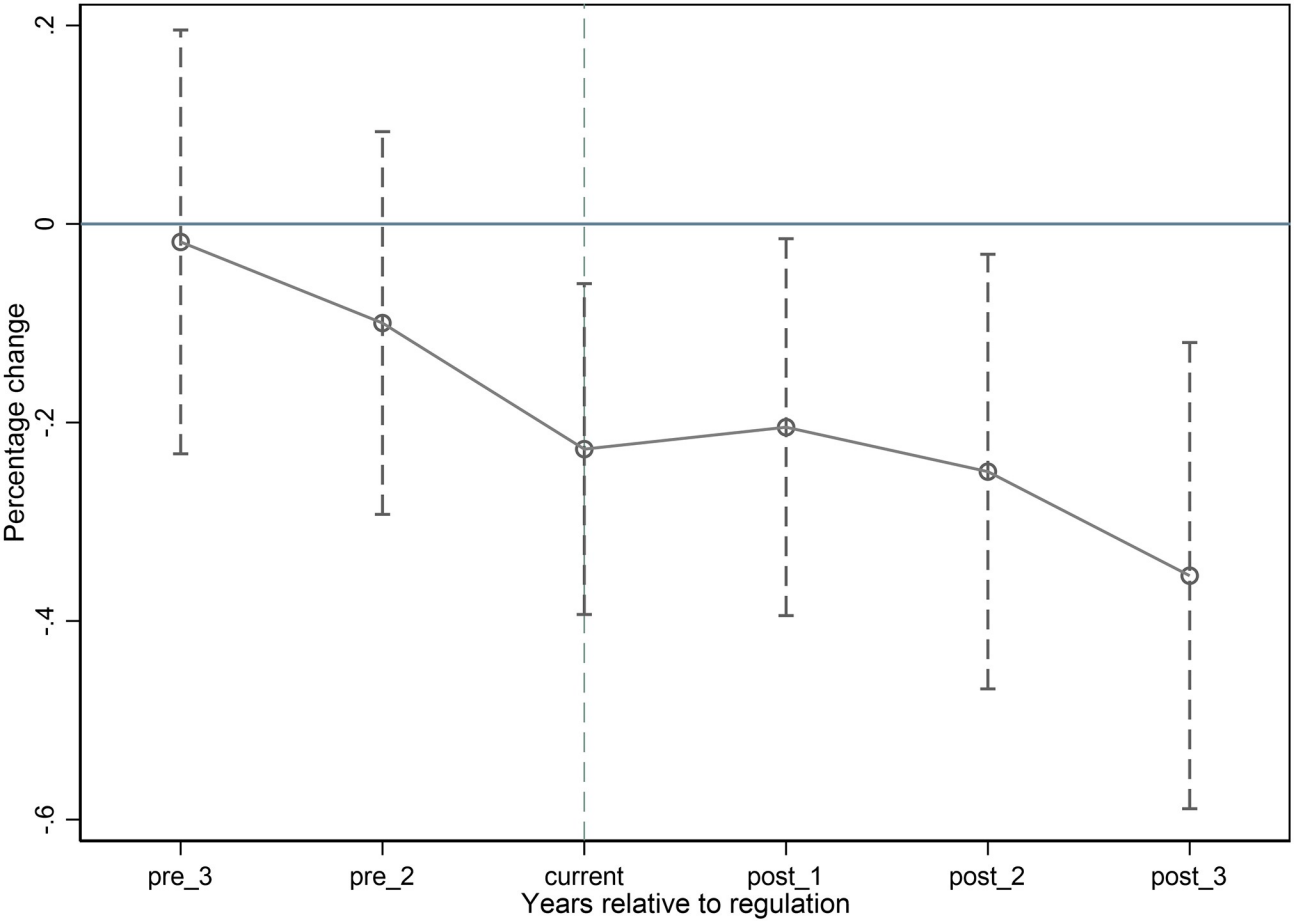
Parallel trend test.

The DID method needs to satisfy the parallel trend assumption. That is, the trend of Strategy in the treated group is not statistically significant different from the trend in the control group, while there is a significant difference in the trends of the two after the policy. Refer to Moser and Voena [44], new time dummy variables are set respectively: from 2 years before the policy to 3 years after the intervention (the dummy variable for time in the first year before the policy, as the baseline variable, has been dropped), multiply the new time dummy variable by the dummy variable stand for the high-polluting companies, then it is incorporated into the DID regression model. The regression results are plotted in Fig 1. From Fig 1, we can see that before the Green Credit Guidelines are issued, there is no significant difference between the market expansion strategies of the control group and the treated group. However, the market expansion strategy tendency of the treated group is significantly lower than that of the control group from the year the Guidelines are issued. This shows that the DID model in this paper satisfies the assumption of parallel trend testing. The text continues here.

We also performed a placebo test to exclude other factors. We consider a possibility: the decline in the aggressiveness of enterprise market expansion strategy is caused by the decline of China’s economic growth, rather than the promulgation of Green Credit Guidelines. To eliminate this potential possibility, we constructed a virtual policy occurrence time before the actual policy occurrence (before 2012). Columns 1 and 2 of Table 5 are the regression results using did two-way fixed effect when the virtual policy time is 2010 and 2011 respectively. We can see that when the construction time of the virtual policy is 2010 and 2011, the coefficient of the interaction term is not significant. Further, we split the sample into 2008–2012 and 2013–2015, and assume that there are two virtual exogenous shocks in 2010 and 2014 respectively. We retest whether the market expansion strategy of high polluting enterprises has changed after the virtual exogenous impact. The results are listed in columns (3) and (4) of Table 5 respectively. The two exogenous virtual shocks still have no significant impact on the market expansion strategy of high pollution enterprises. The results in Table 5 show that there are no other time factors driving the benchmark results in this paper.

**Table 5:** Placebo test.

	(1)	(2)	(3)	(4)
	policy = 2010	policy = 2011	2008–2012	2013–2015
*Pollute*×*Policy*	-0.0105	-0.0263	0.0460	-0.2747
	(0.047)	(0.049)	(0.050)	(0.420)
*size*	0.4579***	0.4578***	0.4981***	0.2032*
	(0.045)	(0.045)	(0.067)	(0.117)
*age*	-0.9854***	-0.9834***	-0.1639***	0.0319
	(0.268)	(0.268)	(0.017)	(0.420)
*leverage*	0.9617***	0.9589***	0.7290***	0.8864**
	(0.158)	(0.158)	(0.224)	(0.378)
*ROE*	1.4054***	1.4038***	0.7586***	-0.3866
	(0.117)	(0.117)	(0.140)	(0.288)
*SOE*	-0.0659	-0.0660	0.1594	-0.6674
	(0.117)	(0.117)	(0.144)	(0.471)
*Top5Hold*	0.0154***	0.0155***	0.0077**	0.0012
	(0.002)	(0.002)	(0.004)	(0.006)
*board*	0.0089	0.0088	-0.0141	0.0283
	(0.016)	(0.016)	(0.021)	(0.043)
*Indpos*	-0.4619**	-0.4658**	-0.3931	-0.1101
	(0.206)	(0.206)	(0.279)	(0.834)
*Indep*	-0.2301	-0.2299	-0.2778	0.1639
	(0.400)	(0.400)	(0.521)	(0.977)
*Mgage*	-0.0358***	-0.0358***	-0.0144	0.0202
	(0.011)	(0.011)	(0.015)	(0.031)
*Constant*	1.8525	1.8363	-6.0324***	-3.1714
	(2.418)	(2.419)	(1.431)	(5.908)
*Observations*	7,272	7,272	4,062	3210
*R-squared*	0.118	0.118	0.084	0.051
*Number of stkcd*	1,727	1,727	1,221	1,577
*Firm*&*Year*	YES	YES	YES	YES

Note:

***, ** and * represent significance levels of 1%, 5% and 10%, respectively.

### 5.4 Robustness test

To ensure the robustness and reliability of the research conclusions, we replace the proxy variables, change the sample, and do PSM-DID (propensity score matching-DID).

1. Replace the variables that measure the market expansion strategy. In Table 4, we calculate the average value of the market expansion tendency of each company in the past five years, rank them from small to large by industry-year, and divide them into six groups to measure the market expansion strategy. Here, we measure the market expansion strategy according to the market share of enterprises in the future. That is, we use the market position (*F*. *Indpos*) and sales scale (*F*. *ln(revenue)*) of enterprises in the next period to measure the market expansion strategy of enterprises in the current period. The definition of market position (*lndpos*) is shown in Table 1. The sales scale of an enterprise is measured by the logarithm of its operating income. The results are listed in columns (1) and (2) of Table 6, respectively. The coefficient of *Pollute* × *Policy* is still significantly negative.
2. After the financial crisis in 2008, China launched a series of economic stimulus policies, such as the implementation of the "four trillion" fiscal stimulus policy, the reduction of loan interest rates for five times and deposit interest rates for four times. In order to eliminate the potential impact of macroeconomic factors on the enterprise’s market expansion strategy, we add the SHIBOR (Shanghai interbank offered rate) as the control variable and re estimated formula 2, shown in column (3) of Table 6. The coefficient of *Pollute* × *Policy* is significantly negative, hypothesis 1 holds. The impact of interest rate on the company’s market expansion strategy is also significantly negative, indicating the looser the monetary policy, the more aggressive market expansion strategy is.
3. We also try to eliminate the interference of other factors on causal identification in this paper. We consider such a situation: the market expansion strategy of high pollution enterprises is different from that of non-high pollution enterprises. This difference may not be brought about by the green credit policy, but an inevitable choice in the process of enterprise development. Since it is mainly up to the management to decide whether the company will carry out an aggressive market expansion strategy, we use the PSM-DID to try to match individuals similar to the management indicators of high polluting enterprises in non-high polluting enterprises as the control group, and then conduct did test. The regression results are listed in column (4) of Table 6. we use the propensity matching score method (PSM) to find the non-high pollution enterprises, which match the control group in non-high pollution enterprises as much as possible, whose management indicators are similar to those of highly polluting enterprises, and take these matched non-highly polluting enterprises as the control group. We re regressed formula 2 with the new control group and treated group. The regression results are listed in column (4) of Table 6. The conclusion of this paper is robust: the promulgation of Green Credit Guidelines inhibits the market expansion tendency of high pollution enterprises.

**Table 6:** Robustness test.

	(1)	(2)	(3)	(4)
	*F*.*IndPos*	*F*. *ln(revenue)*	2009–2015	PSM-DID
*Pollute*×*Policy*	-0.0185***	-0.1332***	-0.1638***	-0.1682***
	(0.004)	(0.029)	(0.062)	(0.062)
*size*	0.0292***	0.4370***	0.4515***	0.4515***
	(0.003)	(0.021)	(0.046)	(0.046)
*age*	0.0288	-0.3038	-0.9614***	-0.9614***
	(0.033)	(0.232)	(0.268)	(0.268)
*leverage*	0.0025	0.5232***	1.0415***	1.0415***
	(0.010)	(0.070)	(0.162)	(0.162)
*ROE*	0.0401***	0.3128***	1.3651***	1.3651***
	(0.008)	(0.054)	(0.124)	(0.124)
*SOE*	0.0137*	0.0789	-0.0870	-0.0870
	(0.007)	(0.051)	(0.122)	(0.122)
*Top5Hold*	0.0004***	0.0006	0.0154***	0.0154***
	(0.000)	(0.001)	(0.002)	(0.002)
*board*	-0.0022**	-0.0027	0.0067	0.0067
	(0.001)	(0.007)	(0.017)	(0.017)
*Indpos*		0.4075***	-0.4820**	-0.4820**
		(0.105)	(0.230)	(0.230)
*Indep*	0.0791***	-0.3809**	-0.1148	-0.1148
	(0.023)	(0.166)	(0.417)	(0.417)
*Mgage*	0.0005	0.0042	-0.0345***	-0.0345***
	(0.001)	(0.004)	(0.011)	(0.011)
*Rate*			-13.8344***	
			(4.350)	
*Constant*	-0.9071***	14.0922***	57.5534***	1.6017
	(0.295)	(2.084)	(19.811)	(2.423)
*Observations*	4,872	4,872	7,272	7,018
*R-squared*	0.141	0.429	0.116	0.116
*Number of stkcd*	1,303	1,303	1,727	1,723
*Firm*&*Year*	YES	YES	YES	YES

Note:

***, ** and * represent significance levels of 1%, 5% and 10%, respectively.

## 6. Discussion

### 6.1 Mechanism analysis

The above results show that the promulgation of green credit policy can effectively inhibit the market expansion strategy of high pollution enterprises. What is the specific mechanism of this inhibition? The previous analysis shows that the green credit policy will increase the financing cost of high polluting enterprises and encourage enterprises to carry out emission reduction R & D, so as to reduce the aggressive market expansion strategy in the short term. These two mechanisms will be tested separately below.

### Financing constraints

Similar to Peng et al. [31], we believe that it is more difficult for highly polluting enterprises to obtain bank loans after the promulgation of Green Credit Guidelines. The guidelines increase the external financing cost of highly polluting enterprises.

We first measure the financial cost of an enterprise by the ratio of its financial expenses to its operating income. We test whether the promulgation of Green Credit Guidelines increases the external financing cost of high polluting enterprises according to formula 3. The regression results are listed in column 1 of Table 7. The coefficient of *Pollute* × *Policy* to cost is significantly positive, which shows that the promulgation of Green Credit Guidelines increases the financial cost of high polluting enterprises.

**Table 7:** Financing constraint perspective.

	(1)	(2)	(3)	(4)	(5)
	*cost*	High cash flow	Low cash flow	High KZ	Low KZ
*Pollute*×*Policy*	0.0052***	-0.2026**	0.0556	-0.0181	-0.2875***
	(0.002)	(0.088)	(0.103)	(0.099)	(0.092)
*size*	0.0032***	0.4771***	0.4605***	0.3390***	0.5339***
	(0.001)	(0.072)	(0.070)	(0.069)	(0.072)
*age*	0.0082	-0.2689	-1.0455***	-0.7985	-0.7290**
	(0.007)	(0.715)	(0.315)	(0.555)	(0.358)
*leverage*	0.0700***	0.6233**	1.2322***	1.3753***	0.9550***
	(0.004)	(0.252)	(0.244)	(0.247)	(0.252)
*ROE*	-0.0135***	1.4849***	1.3245***	1.7475***	1.0744***
	(0.003)	(0.222)	(0.166)	(0.199)	(0.162)
*SOE*	-0.0046	0.1460	-0.0881	0.1723	0.0491
	(0.003)	(0.189)	(0.172)	(0.162)	(0.200)
*Top5Hold*	-0.0000	0.0174***	0.0162***	0.0194***	0.0122***
	(0.000)	(0.003)	(0.004)	(0.003)	(0.004)
*board*	0.0002	0.0048	0.0141	-0.0411*	0.0495*
	(0.000)	(0.023)	(0.027)	(0.023)	(0.026)
*Indpos*	0.0219**	-0.5486	0.2271	0.5236	-1.0219
	(0.010)	(0.595)	(0.641)	(0.598)	(0.632)
*Indep*	-0.0001	-0.0073	-0.0390**	-0.0214	-0.0367**
	(0.000)	(0.015)	(0.017)	(0.016)	(0.017)
*Mgage*	-0.0129**	-0.7056**	-0.0794	-0.8714**	-0.6566**
	(0.005)	(0.293)	(0.346)	(0.359)	(0.294)
*Constant*	-0.1473**	-5.6658	2.1234	1.8793	-1.8997
	(0.060)	(5.980)	(3.072)	(4.769)	(3.379)
*Observations*	7,272	3,641	3,631	3,642	3,630
*R-squared*	0.096	0.126	0.119	0.129	0.114
*Number of stkcd*	1,727	1,292	1,334	1,249	1,255
*Firm*&*Year*	YES	YES	YES	YES	YES

Note:

***, ** and * represent significance levels of 1%, 5% and 10%, respectively.

Corporate strategy can be regarded as the rational choice or reasonable action of the company to create earnings for shareholders in the uncertain market environment. Enterprises with high cash flow and low financing constraints are more likely to adopt aggressive market expansion. The promulgation of the Green Credit Guidelines, an exogenous shock, increases the financing difficulty and cost of high-polluting companies. The Guidelines undoubtedly has a greater impact on these companies with more aggressive market expansion. The impact of this guide on companies with defensive market expansion propensity may be smaller or even insignificant. Therefore, we divided the sample into sub-samples according to the level of cash flow and the level of KZ index and re-regressed Formula 2.

We use the ratio of cash flow generated from operating activities to total assets to measure the size of corporate cash flow. According to whether the size of the company’s cash flow is higher than the median of the same industry in the same year, the sample is divided into two sub-samples of high cash flow and low cash flow. The regression results are listed in columns (2) to (3) of Table 7.

The calculation method of the KZ indicator is based on Kaplan and Zingales [45]: calculate the net operating cash flow/total assets, cash dividends/total assets, cash holdings/total assets, asset-liability ratio and Tobin’s Q value respectively. KZ = -1.001909 * OCF/Asset + 3.139193 * Lev-39. 3678 *Dividends /Asset-1.314759 * Cash /Asset + 0.2826389 * Tobin’s Q. According to whether the company’s KZ index is higher than the median of the same industry in the same year, the sample is divided into two sub-samples with high and low KZ indexes. The regression results are listed in columns (4) and (5) of Table 7.

From the regression results, we can see that the promulgation of Green Credit Guidelines significantly inhibits the market expansion tendency of enterprises with high cash flow level and low KZ index, making these companies more conservative in their strategic choices. However, the impact on enterprises with low cash flow holding level and high financing constraints is not significant. This may be related to the defensive market expansion strategy adopted by these enterprises.


$$\text{cos}t_{i,t}=\chi_{0}+\chi_{1}(Pollute_{i}\times Policy_{t})+\chi_{2}control+\lambda_{i}+\upsilon_{t}+\varepsilon_{i,t}$$ (3)

### R & D innovation

The theoretical analysis, the R & D innovation is sustainable in the enterprises with strong R & D capability. Due to the limited innovation resources, the R & D investment is uncertain in enterprises with weak R & D ability, these enterprises’ R & D investment is often related to the subsidy policy and the financial situation of the enterprises themselves [34, 36]. Therefore, we believe that the promulgation of Green Credit Guidelines can better encourage enterprises with strong R & D ability to further invest in R & D about environmental protection, economy and sustainable development.

On the other hand, both increasing R & D innovation and rapidly expanding the market need financial support. Therefore, enterprises that prefer R & D innovation strategy will appropriately reduce their market expansion tendency to ensure sufficient funds to support R & D. In other words, Green Credit Guidelines have a more significant inhibitory effect on the market expansion strategy of enterprises with strong R & D ability.

Based on the above two analysis, we divide enterprises into two sub samples with strong R & D capability and weak R & D capability, and re estimate formula 2. We believe that the amount of R & D investment can measure the R & D capacity of enterprises to a certain extent. Therefore, the R & D capacity is measured by the proportion of intangible assets in total assets and the proportion of R & D expenses in total costs. Column (1) of Table 8 shows the sub sample regression results that the proportion of intangible assets in total assets is higher than the median of the same industry in the same year, and column (2) of Table 8 shows the sub sample regression results that are lower than the median of the industry. The sub sample regression results of the proportion of R & D expenses in the total cost higher than the median of the same industry in the same year are listed in column (3) of Table 8, and the sub sample regression results lower than the median of the same industry in the same year are listed in column (4) of Table 8.

**Table 8:** R & D perspective.

	(1)	(2)	(3)	(4)
	High intangible assets	low intangible assets	High R & D expenses	low R & D expenses
*Pollute*×*Policy*	-0.2685***	-0.0051	-0.2883***	-0.0517
	(0.091)	(0.093)	(0.089)	(0.088)
*size*	0.4489***	0.4656***	0.5330***	0.4518***
	(0.070)	(0.071)	(0.066)	(0.068)
*age*	-0.5664	-0.9526***	-0.8240**	-1.3964***
	(0.762)	(0.318)	(0.363)	(0.407)
*leverage*	0.4731**	1.3820***	0.5640**	1.2872***
	(0.234)	(0.239)	(0.230)	(0.225)
*ROE*	1.1187***	1.2885***	1.3747***	1.0287***
	(0.176)	(0.170)	(0.175)	(0.162)
*SOE*	0.0887	-0.1349	0.1816	-0.3082*
	(0.185)	(0.179)	(0.162)	(0.171)
*Top5Hold*	0.0103***	0.0210***	0.0081**	0.0156***
	(0.003)	(0.004)	(0.003)	(0.003)
*board*	-0.0263	0.0205	-0.0319	0.0175
	(0.023)	(0.024)	(0.024)	(0.022)
*Indpos*	-0.6269*	-0.3251	-0.7282**	-0.2243
	(0.322)	(0.341)	(0.351)	(0.271)
*Indep*	-0.1533	0.2711	-0.6386	0.5752
	(0.573)	(0.613)	(0.596)	(0.540)
*Mgage*	-0.0580***	-0.0083	-0.0675***	-0.0182
	(0.016)	(0.016)	(0.016)	(0.015)
*Constant*	0.3816	-0.5526	1.2148	4.4153
	(6.362)	(3.113)	(3.325)	(3.672)
*Observations*	3,641	3,631	3,646	3,626
*R-squared*	0.139	0.109	0.146	0.113
*Number of stkcd*	1,102	1,109	1,109	1,101
*Firm*&*Year*	YES	YES	YES	YES

Note:

***, ** and * represent significance levels of 1%, 5% and 10%, respectively.

From the regression results, we can see that the promulgation of Green Credit Guidelines significantly inhibits the market expansion tendency of high polluting enterprises with strong R & D and innovation ability. This shows that the green credit policy has stimulated high polluting enterprises to further increase investment in green innovation. Hypothesis 3 holds.

We can also see from Table 8 that the industry status of enterprises with strong innovation ability is negatively correlated with the market expansion tendency. This shows that enterprises with strong innovation ability will choose to shift from expanding the production and sales scale of products to expanding green technology after the market share reaches a certain degree. However, this negative correlation is not significant in enterprises with low innovation ability, which further verifies that the green innovation investment of enterprises with weak innovation ability is random [33].

In addition, in Table 8, in enterprises with strong innovation ability, there is a significant negative correlation between managements’ age and market expansion tendency, which is not significant in enterprises with weak innovation ability. This shows that in enterprises with strong innovation ability, the older the managements are, the more attention is paid to product creativity and product quality, rather than the simple and rough growth of sales scale.

### 6.2. Further discussion

#### 6.2.1 Heterogeneity analysis

After the global financial crisis, the $4 trillion stimulus plan led state-owned enterprises to obtain more credit resources than non-state-owned enterprises, exacerbated the difference in resource availability between state-owned enterprises and non-state-owned enterprises [46]. In addition, state-owned enterprises can obtain implicit guarantees from the government [47]. Highly polluting state-owned enterprises may be less affected than highly polluting non-state-owned enterprises, in the face of financing constraints after the promulgation of Green Credit Guidelines. Therefore, we divide the sample into two sub samples of state-owned enterprises and non-state-owned enterprises, and re regress formula 2. The results are listed in columns (1) and (2) of Table 9. The promulgation of Green Credit Guidelines significantly reduces the market expansion tendency of non-state-owned enterprises, but the impact on state-owned enterprises is not significant. This confirms our view that state-owned enterprises are less constrained by the green credit policy because of their enterprise nature.

**Table 9:** Heterogeneity analysis.

	(1)	(2)	(3)	(4)
	SOE	Non-SOE	Option Inspiration	Non-Option Inspiration
*Pollute*×*Policy*	-0.1089	-0.2310**	-0.1780	-0.1720***
	(0.077)	(0.106)	(0.219)	(0.064)
*size*	0.5175***	0.3716***	0.5962***	0.4409***
	(0.063)	(0.069)	(0.161)	(0.047)
*age*	-1.2879***	-0.5353	-0.6513	-1.0345***
	(0.344)	(0.678)	(0.666)	(0.291)
*leverage*	1.0486***	0.7734***	1.3715***	0.9101***
	(0.217)	(0.236)	(0.520)	(0.165)
*ROE*	1.4191***	1.3138***	1.9932***	1.3526***
	(0.146)	(0.204)	(0.420)	(0.122)
*SOE*			0.3411	-0.1495
			(0.261)	(0.130)
*Top5Hold*	0.0115***	0.0235***	0.0188***	0.0155***
	(0.003)	(0.003)	(0.007)	(0.002)
*board*	0.0108	-0.0079	0.0164	0.0078
	(0.020)	(0.028)	(0.044)	(0.017)
*Indpos*	0.5892	-1.5903**	-0.4192	-0.1566
	(0.509)	(0.685)	(1.087)	(0.428)
*Indep*	-0.0362**	-0.0163	-0.0614*	-0.0309***
	(0.014)	(0.016)	(0.032)	(0.011)
*Mgage*	-0.4651*	-0.2119	-0.5132	-0.4293*
	(0.257)	(0.465)	(0.473)	(0.228)
*Constant*	4.2464	-1.3056	-3.9491	2.5827
	(3.556)	(4.590)	(5.650)	(2.649)
*Observations*	4,329	2,943	602	6,670
*R-squared*	0.126	0.115	0.219	0.115
*Number of stkcd*	882	908	144	1,583
*Firm*&*Year*	YES	YES	YES	YES

Note:

***, ** and * represent significance levels of 1%, 5% and 10%, respectively.

The promulgation of green credit guidelines is bad for high polluting enterprises. The Guides declare China’s determination to achieve high-quality and sustainable economic development in the future. Therefore, the Guides will increase the managements’ performance pressure of high pollution enterprises. In order to cope with the pressure of market performance and avoid the loss of potential shareholders’ wealth, the management of enterprises with equity incentive may sacrifice some long-term projects [48, 49] in exchange for short-term profits. In order to cater to shareholders, these enterprises may further adopt aggressive market expansion strategies rather than green R & D. On the contrary, Green Credit Guidelines have less impact on the performance pressure of enterprises without equity incentive. The management of these enterprises may pay more attention to the sustainability of future development and the optimization of product quality, and try their best to adjust the enterprise strategic objectives to make corporate strategies consistent with the national strategic. These enterprises may focus on green R & D innovation. Therefore, we divide the sample into two sub samples: with and without equity incentive, and re regress formula 2. The results are listed in columns (3)—(4) of Table 9. The promulgation of Green Credit Guidelines significantly inhibits the market expansion strategy of companies without equity incentive. This shows that our reasoning is tenable: enterprises without equity incentive will focus on sustainable and green R & D, so they will reduce market expansion in the short term.

#### 6.2.2 Economic effects of Green Credit Guidelines

This paper further examines the economic effects of Green Credit Guidelines on corporate value from three aspects: corporate image, profitability and risk. The regression model is formula 4. We measure the corporate image by negative media sentiment (NEG) and the average earnings per share predicted by analysts (FEPS). Negative media sentiment (NEG) is the ratio between the number of negative financial news and the total amount of financial news of the company in that year. We measure the profitability of the company by return on total assets (*ROA*) and return on equity (*ROE*). We measure corporate risk by ROE’s volatility (*roe_sd*) and stock price collapse risk (*Ncskew*). ROE’s volatility (*roe_sd*) is the rolling standard deviation of the company’s ROE over three years. Referring to the research of Hutton et al. [50] and Kim et al. [51], we use the following methods to measure the risk of stock price collapse (*Ncskew*). First, we calculate the idiosyncratic weekly stock return adjusted by the market return *ε*_*i*,*t*_, according to formula 5. *r*_*i*,*t*_ is the reinvestment return of company *i* in week *t*, and *r*_*m*,*t*_ is the weighted average market return of all A-share companies in week *t*. The residual term *ε*_*i*,*t*_ is a unique rate of return that cannot be explained by the market rate of return in time *t*. *W*_*i*,*t*_ = ln(1 + *ε*_*i*,*t*_). Then, we calculate *W*_*i*,*t*_, and construct the index *Ncskew* to measure the risk of stock price collapse according to formula 6. The regression results of the Green Credit Guidelines on economic effects are shown in Table 10.


$$Effect_{i,t}=\beta_{0}+\beta_{1}(Pollute_{i}\times Policy_{t})+\beta_{2}control+\lambda_{i}+\upsilon_{t}+\varepsilon_{i,t}$$ (4)

$$r_{i,t}=\alpha_{0}+\alpha_{1}r_{m,t-2}+\alpha_{2}r_{m,t-1}+\alpha_{3}r_{m,t}+\alpha_{4}r_{m,t+1}+\alpha_{5}r_{m,t+2}+\varepsilon_{i,t}$$ (5)

$$Ncskew_{i,t}=-n{\left(n-1\right)}^{3/2}\sum W_{i,t}^{3}/((n-1)(n-2){\left(\sum W_{i,t}^{2}\right)}^{3/2})$$ (6)

**Table 10:** The economic effects of Green Credit Guidelines.

	Corporate image		Profitability		Risk	
	Neg	Feps	Roa	Roe	Roe_sd	Ncskew
*Pollute*×*Policy*	0.0282***	-0.0977***	-0.0115***	-0.0239***	0.0362	0.0345
	(0.008)	(0.020)	(0.003)	(0.008)	(0.024)	(0.044)
*size*	-0.0326***	0.1580***	-0.0107***	-0.0296***	-0.1432***	-0.0404
	(0.006)	(0.015)	(0.002)	(0.006)	(0.017)	(0.032)**
*age*	0.0104	-0.0642	0.0105	0.0107	0.0601	-0.4930**
	(0.033)	(0.088)	(0.011)	(0.035)	(0.105)	(0.222)
*leverage*	0.0596***	-0.2547***	-0.0067	0.0508**	0.5955***	0.0885
	(0.019)	(0.053)	(0.006)	(0.020)	(0.060)	(0.112)
*ROE*	-0.1667***	1.0487***				0.0541
	(0.014)	(0.041)				(0.085)
*SOE*	0.0053	-0.0541	-0.0126***	-0.0172	-0.0977**	-0.0417
	(0.014)	(0.039)	(0.005)	(0.015)	(0.046)	(0.081)
*Top5Hold*	-0.0000	-0.0010	0.0008***	0.0019***	0.0033***	-0.0006
	(0.000)	(0.001)	(0.000)	(0.000)	(0.001)	(0.002)
*board*	0.0006	-0.0022	0.0015**	0.0060***	0.0009	-0.0139
	(0.002)	(0.005)	(0.001)	(0.002)	(0.006)	(0.011)
*Indpos*	0.0402	-0.2702**	-0.0024	0.0224	-0.1560	-0.0491
	(0.049)	(0.133)	(0.016)	(0.053)	(0.156)	(0.277)
*Indep*	0.0001	0.0057	-0.0002	-0.0028**	-0.0034	0.0060
	(0.001)	(0.003)	(0.000)	(0.001)	(0.004)	(0.007)
*Mgage*	-0.0183	0.1473**	0.0282***	0.0889***	0.1345*	-0.1688
	(0.025)	(0.067)	(0.008)	(0.027)	(0.080)	(0.140)
*Constant*	0.8396***	-2.4040***	0.1539	0.5826*	2.5675***	4.4688**
	(0.298)	(0.791)	(0.097)	(0.317)	(0.946)	(2.002)
*Observations*	7,266	6,522	7,065	7,065	7,272	6,603
*R-squared*	0.109	0.204	0.080	0.049	0.033	0.056
*Number of stkcd*	1,727	1,685	1,705	1,705	1,727	1,602
*Firm&Year*	YES	YES	YES	YES	YES	YES

Note:

***, ** and * represent significance levels of 1%, 5% and 10%, respectively.

First of all, the promulgation of Green Credit Guidelines undoubtedly damages the corporate image of highly polluting enterprises. As can be seen from Table 10, the proportion of negative financial news of high polluting enterprises increased by 2.82% compared with non-high polluting enterprises, after the promulgation of the Green Credit Guidelines. Analysts’ earnings per share forecast for highly polluting enterprises decreased by 0.0977, compared with non-highly polluting enterprises. This shows that the promulgation of the Guidelines has increased the public’s attention to sustainability, low carbon and emission reduction. The media and professional institutions have increased the supervision of enterprises that violate the concept of green development. After the promulgation of Green Credit Guidelines, the corporate image of highly polluting enterprises is damaged.

The promulgation of Green Credit Guidelines has also reduced the profitability of highly polluting enterprises. Guidelines increase the cost of borrowing from banks for highly polluting enterprises and encourage enterprises to increase the R & D of green and sustainable projects. Both channels reduce the profitability of highly polluting enterprises. The results in columns (3) and (4) of Table 10 confirm our view.

The promulgation of Green Credit Guidelines do not increase the risk of high polluting enterprises. Whether the risk is measured by *roe_sd* or *Ncskew*, the impact of the Guidelines on the risk is not significant, as shown in column (5) and (6) of Table 10. In other words, although the promulgation of Guidelines will damage part of the profitability of high polluting enterprises, it will not increase the risk of enterprises. We believe that market-oriented environmental regulation is an effective and safe environmental protection policy.

## 7. Conclusions

Based on the quasi-natural experiment of the promulgation of China’s Green Credit Guidelines, we empirically investigate the impact of market-oriented environmental regulation on the choice of company’s market expansion strategy, taking A-share listed companies from 2008 to 2015 as a sample. The results show that the promulgation of Green Credit Guidelines improves the financing constraints of high polluting enterprises, encourages high polluting enterprises to increase the R & D of green projects, and then promotes the company’s market expansion strategy to defensive. Further, the effect of the Green Credit Guidelines on firm’s market expansion strategy is more prominent if the firm is non-SOE or without equity incentive. We also show that although the promulgation of the Green Credit Guidelines reduced the profits of highly polluting enterprises, it does not increase their risk of stock price collapse. In sum, we conclude that the Green Credit Guidelines are market based environmental regulations in line with Porter’s hypothesis. This paper reveals the internal relationship between Green Credit Guidelines and corporate behavior from a deeper level, and supplements literatures about the economic effects of market-oriented environmental regulation and corporate strategic management. It provides an academic basis for governments to steadily and effectively promote environmental protection policies.

This study also has some policy implications. First, regulators should actively promote market-oriented environmental regulation. Market-oriented environmental regulation is conducive to promote enterprises to increase the R & D of green projects, protect the environment and achieve sustainable development; It is also conducive to restrict the overly radical business decisions of high pollution enterprises and prevent the occurrence of systemic financial risks. Second, the company’s management should give more consideration to social responsibility and national strategy when deciding the company’s strategy, which will help the company better expand its living space and grasp development opportunities. According to the research conclusion of this paper, if the management does not consider sustainable development and environmental protection when formulating the company’s strategy, it may artificially create obstacles to the survival and development of the company. On the contrary, it will help the management to resolve business risks and ensure the sustainable development of the company. We suggest continued future research on how the green credit policy affects other strategic choices of enterprises, such as talent strategy, M & A strategy or integration strategy, to comprehensively identify green credit policy’s impacts on the transformation of highly polluting enterprises.

In the future, we can continue to study whether the Green Credit Guidelines will have an impact on other strategic choices of highly polluting enterprises, such as diversification strategy and market penetration strategy.

## Supporting information

S1 File (DOCX)
